# How do serum lipid levels change and influence progression-free survival in epithelial ovarian cancer patients receiving bevacizumab treatment?

**DOI:** 10.3389/fonc.2023.1168996

**Published:** 2023-03-29

**Authors:** Xiaoyu Huang, Yong Huang, Ping Li

**Affiliations:** ^1^ Department of Chinese Integrative Medicine Oncology, The First Affiliated Hospital of Anhui Medical University, Hefei, China; ^2^ First Clinical Medical College, Anhui Medical University, Hefei, China; ^3^ Department of Medical Oncology, The Second People’s Hospital of Hefei, Hefei, China

**Keywords:** epithelial ovarian cancer, serum lipid level, change, influence, progression free survival, bevacizumab treatment

## Abstract

**Background:**

This study aimed to investigate how serum lipid levels affect epithelial ovarian cancer (EOC) patients receiving bevacizumab treatment and to develop a model for predicting the patients’ prognosis.

**Methods:**

A total of 139 EOC patients receiving bevacizumab treatment were involved in this study. Statistical analysis was used to compare the median and average values of serum lipid level variables between the baseline and final follow-up. Additionally, a method based on machine learning was proposed to identify independent risk factors for estimating progression-free survival (PFS) in EOC patients receiving bevacizumab treatment. A PFS nomogram dividing the patients into low- and high-risk categories was created based on these independent prognostic variables. Finally, Kaplan–Meier curves and log-rank tests were utilized to perform survival analysis.

**Results:**

Among EOC patients involved in this study, statistical analysis of serum lipid level variables revealed a substantial increase in total cholesterol, triglycerides, apolipoprotein A1, and free fatty acids, and a significant decrease in apolipoprotein B from baseline to final follow-up. Our method identified FIGO stage, combined chemotherapy regimen, activated partial thromboplastin time, globulin, direct bilirubin, free fatty acids, blood urea nitrogen, high-density lipoprotein cholesterol, and triglycerides as risk factors. These risk factors were then included in our nomogram as independent predictors for EOC patients. PFS was substantially different between the low-risk group (total score < 298) and the high-risk group (total score ≥ 298) according to Kaplan–Meier curves (P < 0.05).

**Conclusion:**

Serum lipid levels changed variously in EOC patients receiving bevacizumab treatment. A prediction model for PFS of EOC patients receiving bevacizumab treatment was constructed, and it can be beneficial in determining the prognosis, selecting a treatment plan, and monitoring these patients’ long-term care.

## Introduction

Ovarian cancer represents a significant threat to women’s health, as it is one of the most fatal gynecological malignancies and ranks as the sixth leading cause of cancer-related death among women (1, 2). Epithelial ovarian cancer (EOC) is the most common subtype, accounting for 90% of ovarian cancer cases. Treatment for newly diagnosed EOC routinely involves cytoreductive surgery and platinum-based chemotherapy (3). However, most of them relapse within three years after receiving standard therapy (4). Additionally, the progression-free survival (PFS) tends to decline with each subsequent recurrence as patients undergo further treatments (5). As a consequence, discovering novel therapeutic approaches to improve their prognosis is of utmost importance. Inhibitors of angiogenesis offering a more precise treatment of EOC have been extensively studied. A humanized anti-VEGF monoclonal antibody known as bevacizumab is not only the first actively targeted therapy for EOC, but also the most widely studied anti-angiogenic medication for many types of cancer (6). Attempts to improve standard platinum-based treatment by including bevacizumab have been partially successful in prolonging the PFS of EOC patients, although the effect is only seen in a minority of cases (7, 8). The prognosis of EOC patients receiving bevacizumab treatment is known to be variable, and is typically attributed to several factors including the stage of the disease at diagnosis, the frequency of disease recurrence, and the emergence of drug resistance (9). Considering the high expense, potential toxicity, and limited clinical benefits associated with bevacizumab treatment, it is imperative to comprehensively understand the mechanisms underlying bevacizumab resistance, identify reliable predictive factors, and establish an accurate prediction model.

Bevacizumab resistance is a complex phenomenon, with various metabolic pathways playing an important role in its mechanism. Preclinical studies have suggested that bevacizumab increases intratumoral hypoxia, leading to metabolic reprogramming of fatty acid oxidation and higher levels of free fatty acid absorption. This, in turn, accelerates cancer cell proliferation (10). Additionally, high levels of lipid metabolism in cancer cells indicate a switch from aerobic glycolysis to beta-oxidation and lipogenesis (11, 12), which can promote tumor growth. However, specific changes in various blood lipid levels in real-world EOC patients receiving bevacizumab treatment and nomograms including serum lipid levels to predict PFS of EOC patients are still absent.

To improve the accuracy of PFS prediction for EOCs, Cox regression models have gained popularity as a method for making predictions about the prognosis (13). However, these models demand the expertise of physicians and can be quite time-consuming and labor-intensive. A method using machine learning techniques (14) has been introduced to discover the extremely complex and linear/nonlinear connections between risk factors and a patient’s probability of cancer recurrence (15). In practice, this method has even demonstrated the ability to provide personalized suggestions based on calculated risk (16).

In this study, a total of 139 EOC patients receiving bevacizumab treatment were analyzed. The effects of serum lipid levels on EOC patients receiving bevacizumab treatment were explored, and a prediction model of PFS for these patients was proposed furtherly. The findings of this study indicated changes of serum lipid levels and their influence on PFS in EOC patients receiving bevacizumab treatment.

## Materials and methods

### Patients and assessments

A total of 139 EOC patients receiving bevacizumab treatment between January 2013 and December 2022 at the authors’ institution were retrospectively analyzed. The following were the criteria for selection: 1) histologically verified EOC; 2) at least 18 years of age; 3) Eastern Cooperative Oncology Group (ECOG) level of performance of 0 to 2; 4) normal bone marrow, liver, and kidney functions. Major exclusion criteria included having a history of cancer, pregnancy or lactation, or having a serious coexisting disease.

Age, height, weight, body mass index (BMI), International Federation of Gynecology and Obstetrics (FIGO) stage, ECOG score, and combined chemotherapy regimen (CCR) were recorded as the baseline data of patients. Prior to each cycle of bevacizumab treatment, and subsequently at intervals of two months during the first year, three months during the second year, six months during years three and four, and annually thereafter, clinical evaluations, measurements of blood coagulation parameters, lipid metabolism, and cancer immune responses were conducted. The primary outcome of the study was PFS, calculated from the date of randomization to the date of the first observed indication of cancer progression or death. Progression of the disease was determined in accordance with the Response Evaluation Criteria in Solid Tumors (RECIST) (17) recommendations, based on clinical, radiographic, or symptomatic signs. In addition to PFS, the study also assessed variations in blood lipid levels before and after the randomized date (t1) as well as up to the date of the first sign of disease progression or death, or until December 2022 (t2). Notably, data for this study were updated as of December 2022.

### Statistical analysis

The Wilcoxon signed-rank test or the paired-samples *t-*test was used to compare the median or average values of the serum lipid level variables between t1 and t2. A P-value of 0.05 or less was considered statistically significant.

A method was proposed for predicting PFS and extracting risk factors for patients with EOC who receive bevacizumab treatment. The method consisted of the following steps: First, clinical baseline data such as age, height, and weight of EOC patients receiving bevacizumab treatment were collected and recorded. The progression of ovarian cancer and the corresponding PFS data were extracted and labeled as outcome variables. The collected data underwent data coding, data cleaning, normalization, and other pretreatment operations to obtain processed data. The data set were then divided into a training set and a testing set in an 8:2 ratio, which was used for training and testing significant feature extraction and survival prediction for PFS.

Second, a full-feature classification model was constructed to predict EOC progression after medication. The generalization ability index of the model was evaluated using the classification accuracy of the testing set. The Relief feature selection algorithm (18) was employed to obtain an alternative significant feature set of EOC progression, which was used to extract the significant feature set of EOC progression. A prediction model for EOC survival prognosis was then established based on the extracted significant features.

**Figure 1:**
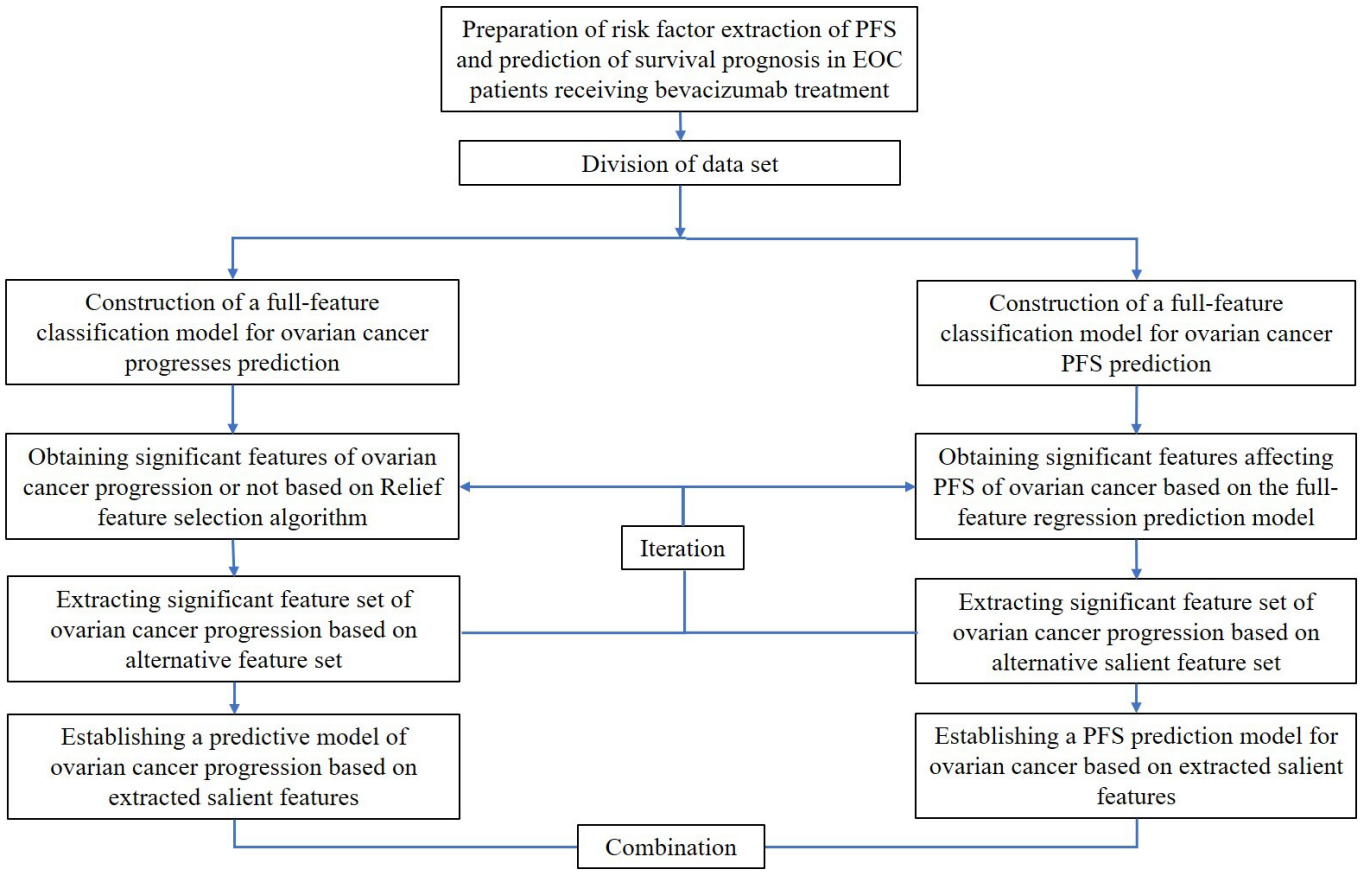
The flowchart of a PFS prediction and risk factors extraction method for EOC patients receiving bevacizumab treatment.

Third, a full-feature regression model for PFS after EOC medication was constructed, and its generalization ability index was evaluated using the coefficient of determination of the testing set. The PFS alternative feature set after EOC medication was obtained based on the total feature regression prediction model, and the PFS significant feature set after bevacizumab treatment was extracted based on the alternative feature set. Using the extracted substantial features, a prediction model of PFS data after EOC treatment was established. Finally, the survival prognosis model and the significant feature extraction and survival prediction model were combined to construct a model for predicting patients’ prognosis. The flowchart of creating the PFS prediction and risk factor extraction method is presented in **Figure 1**.

Light Gradient Boosting Machine (LGBM), Extreme Gradient Boosting (XGBoost), Histogram-Based Gradient Boosting (HGBoost) (19), and Extra-Trees models (20) were developed in the training set to predict PFS for patients with EOC. Receiver operating characteristic (ROC) curves and areas under the curves (AUCs) were used to evaluate the performance of the prediction model, which ranged from 0.50 to 1.0 for our prediction model. The discrimination score ranged from 0.50 to 1.0 in our prediction model, with the latter indicating the highest level of discriminatory capacity. The accuracy of our prediction model was evaluated by determination coefficient (R^2^ score), which measures the proportion of dependent variable variation that can be accounted for by the independent variables through a regression relationship. The higher the R^2^ score, the more accurate the prediction is. Calibration curves were also generated to assess the deviation between the actual and expected outcomes. Improved accuracy of the prediction was indicated by a calibration curve that was closer to the diagonal line.

A nomogram was produced by extracting independent risk factors using the best-performing prediction model. The total score for the nomogram was calculated by assigning scores to each factor that was acquired in accordance with the degree to which the factor affected PFS. Patients were separated into distinct groups (low-risk and high-risk patients) based on the cutoff value determined from the total score of the nomogram. Log-rank analyses and Kaplan–Meier curves were used to assess patient survival rates between the groups. Data analysis and model development were carried out using SPSS 24.0 Software, RStudio, and Python Software Foundation. A P-value of 0.05 or less was considered statistically significant.

## Results

### Patients and characteristics

Among the 139 patients with EOC, 31 remained free of recurrence, while 108 experienced either recurrence or mortality. The study consisted of a training set that comprised 111 EOC patients and a testing set that included 28 EOC patients, with comparable baseline characteristics in both groups. **Table 1** presents the baseline characteristics of the patients in both the training and testing sets.

**Table 1:** Patient and Tumor Characteristics.

Characteristic	Total (n=139), %	Training set (n=111), %	Testing set (n=28), %
Age (years)			
≤60	97(69.78)	77(69.37)	20(71.43)
>60	42(30.22)	34(30.63)	8(28.57)
BMI			
<18	9(6.47)	7(6.30)	2(7.14)
18≤X<24	80(57.55)	64(57.66)	16(57.14)
24≤X<28	46(33.09)	37(33.33)	9(32.14)
≥28	4(2.89)	3(2.71)	1(3.58)
FIGO stage			
Stage I	2(1.45)	2(1.79)	0(0)
Stage II	9(6.47)	7(6.30)	2(7.14)
Stage III	79(56.83)	63(56.76)	16(57.14)
Stage IV	49(35.25)	39(35.13)	10(35.72)
ECOG*			
0	39(28.06)	31(27.93)	8(28.57)
1	88(63.30)	70(63.06)	18(64.29)
2	12(8.64)	10(9.01)	2(7.14)
CCR			
Platinum/paclitaxel	95(68.34)	76(68.47)	19(67.86)
Gemcitabine/Platinum	16(11.51)	13(11.71)	3(10.71)
Others	28(20.15)	22(19.82)	6(21.43)

* ECOG performance status on a scale from 0 to 4, with 0 indicating normal activity, 1 symptomatic but ambulatory self-care possible, 2 ambulatory more than 50% of the time, 3 ambulatory 50% of the time or less and nursing care required, and 4 bedridden and possibly requiring hospitalization.

### Comparison of serum lipid levels

**Table 2** presents a comparison of the mean and median values of serum lipid level variables between t1 and t2 in the entire study cohort. The following serum lipid level variables were included in the analysis: total cholesterol (TC), triglycerides (TG), high-density lipoprotein cholesterol (HDL), non-high-density lipoprotein cholesterol (n-HDL), low-density lipoprotein cholesterol (LDL), very-low-density lipoprotein cholesterol (VLDL), apolipoprotein A1 (APOA1), apolipoprotein B (APOB), lipoprotein-a (LP-a), and free fatty acids (FFA). Most patients exhibited an increase in TC, TG, n-HDL, LDL, VLDL, APOA1, and FFA, while a decrease in HDL-C, APOB, and LP-a was observed following bevacizumab treatment. However, only the increases in TC, TG, APOA1, and FFA, as well as the decrease in APOB, were statistically significant (P < 0.05).

**Table 2:** The relation between changes to serum lipid level variables.

Variables		Mean (SD)	Median	Paired Samples Test/ Wilcoxon Signed Rank Test	
				T/Z	P
TC	t1 t2	4.78 (1.12) 4.98 (1.32)	4.66 4.96	-2.045	0.043
TG	t1 t2	1.66 (0.88) 1.85 (1.04)	1.41 1.48	-3.097	0.001
HDL-C	t1 t2	1.20 (0.29) 1.19 (0.35)	1.16 1.15	-0.036	0.971
n-HDL	t1 t2	3.60 (1.00) 3.68 (1.13)	3.52 3.63	-0.945	0.346
LDL-C	t1 t2	2.98 (0.91) 3.02 (1.08)	2.93 3.00	-0.583	0.561
VLDL	t1 t2	0.63 (0.44) 0.66 (0.46)	0.52 0.54	-1.836	0.066
APOA1	t1 t2	1.23 (0.24) 1.26 (0.28)	1.21 1.24	-2.125	0.034
APOB	t1 t2	1.00 (1.00) 0.96 (0.27)	0.91 0.93	-2.374	0.018
LP-a	t1 t2	302.31 (297.71) 289.17 (319.06)	226 203	-1.588	0.112
FFA	t1 t2	0.37 (0.16) 0.41 (0.20)	0.36 0.41	-2.153	0.031

### Curves of calibration and validation

**Figure 2:**
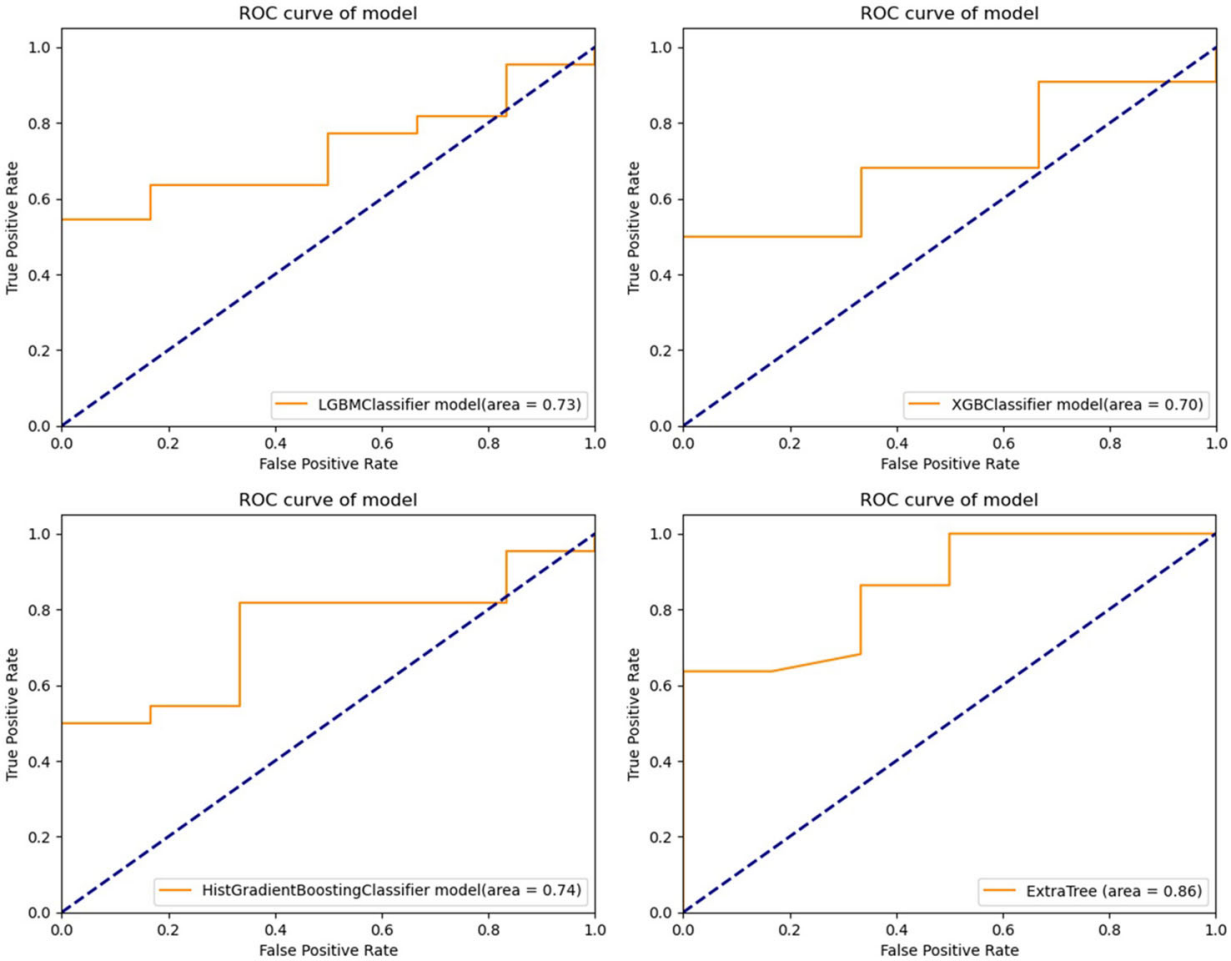
Receiver operating characteristic curves of different prediction models for progression free survival in patients with epithelial ovarian cancer (upper left: LGBM model; upper right: XGBoost model; lower left: HGBoost model; lower right: Extra-Trees model).

**Figure 3:**
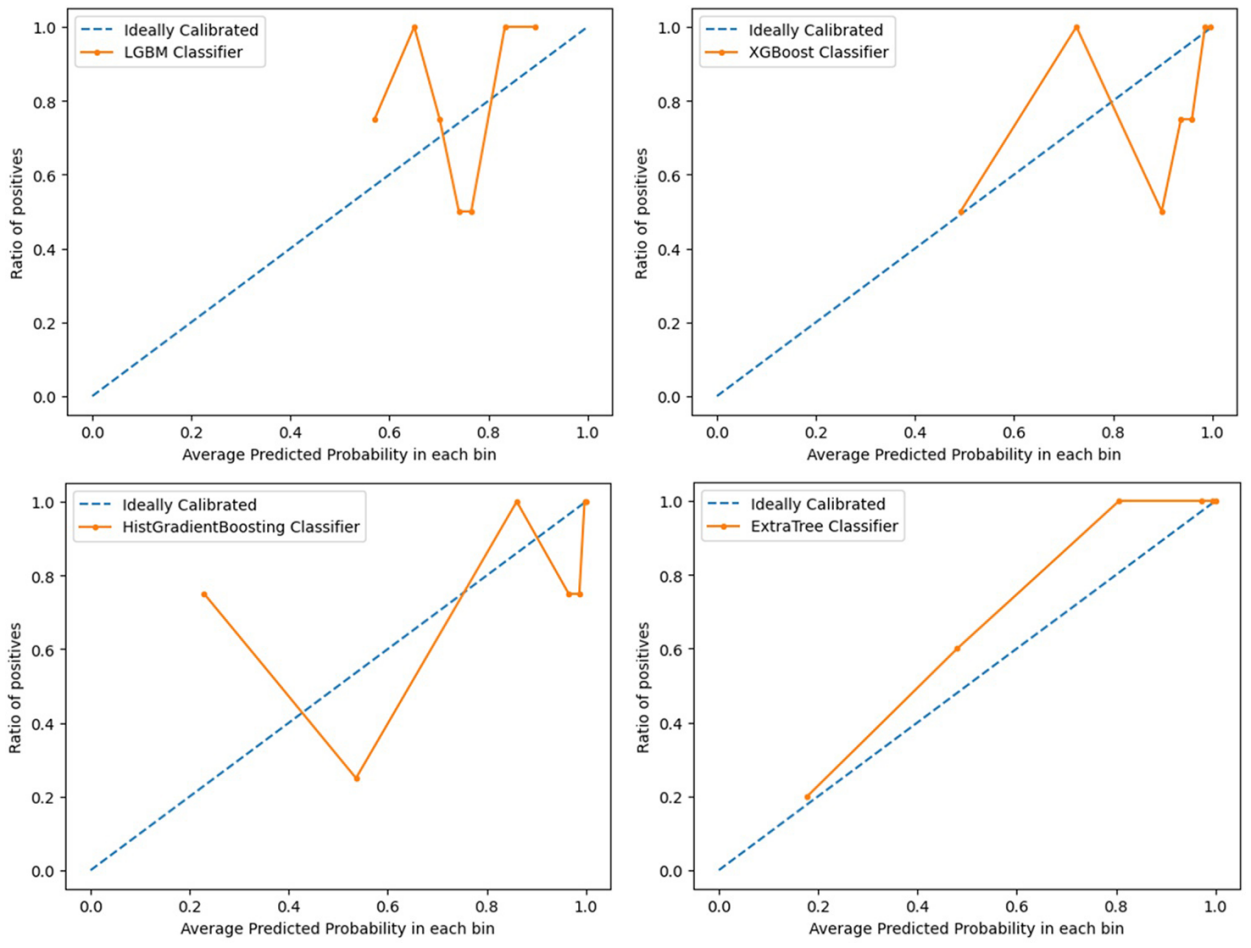
Calibration curves evaluating performance of different prediction models for progression free survival in patients with epithelial ovarian cancer (upper left: LGBM model; upper right: XGBoost model; lower left: HGBoost model; lower right: Extra-Trees model).

Light GBM classifier, XGB classifier, HGBoost Classifier, and Extra-Trees models were created to predict the patients’ PFS. These models’ effectiveness was assessed using ROC curves and AUCs. Calibration curves were also used to evaluate the performance of the models. **Figures 2**, **3** demonstrate how our performance measures clearly preferred the Extra-Trees model for PFS prediction over the other models. The Extra-Trees model was ultimately selected as our PFS prediction model.

### Survival curves

**Figure 4:**
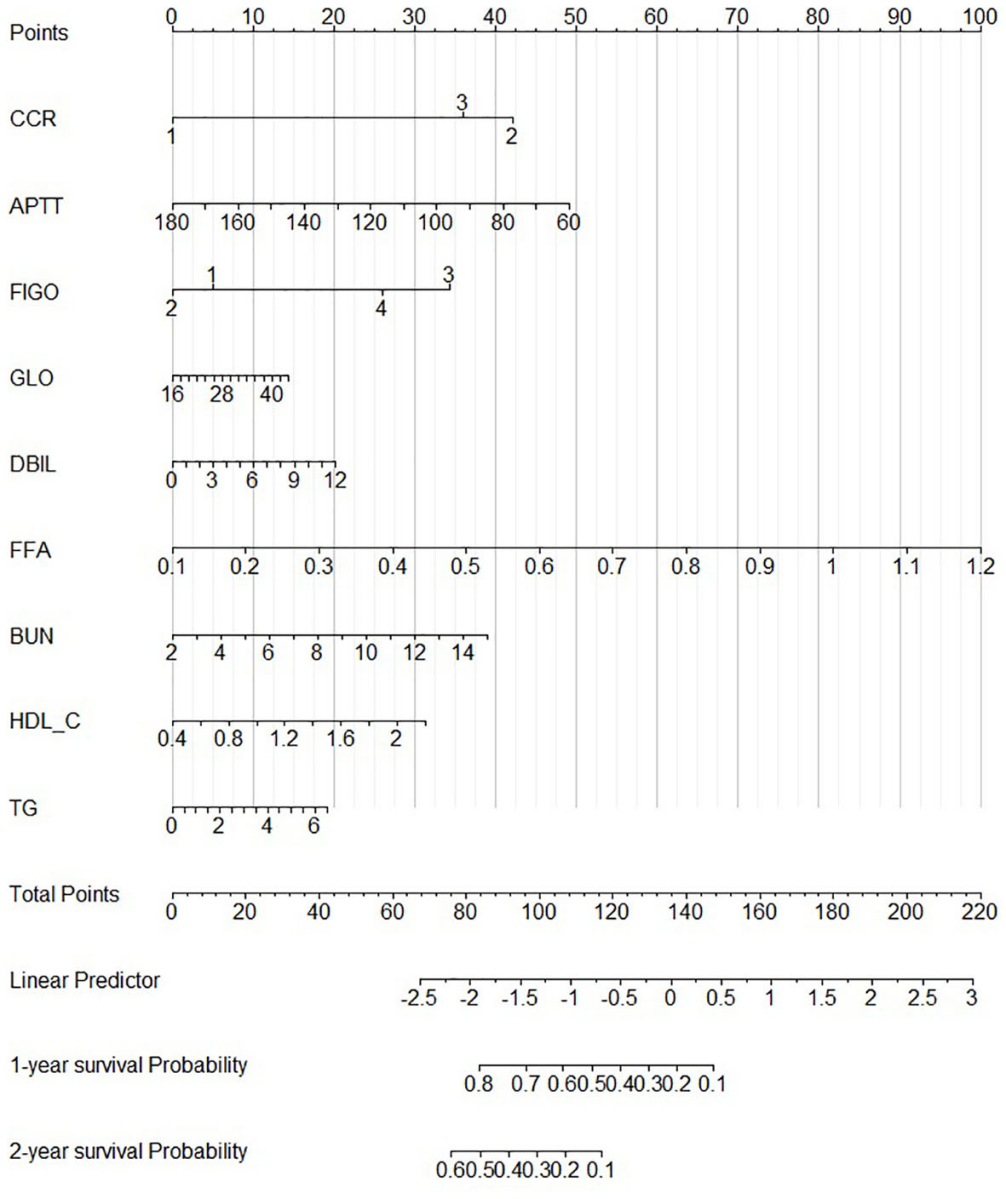
Nomogram for 1- and 2-year PFS.

**Figure 5:**
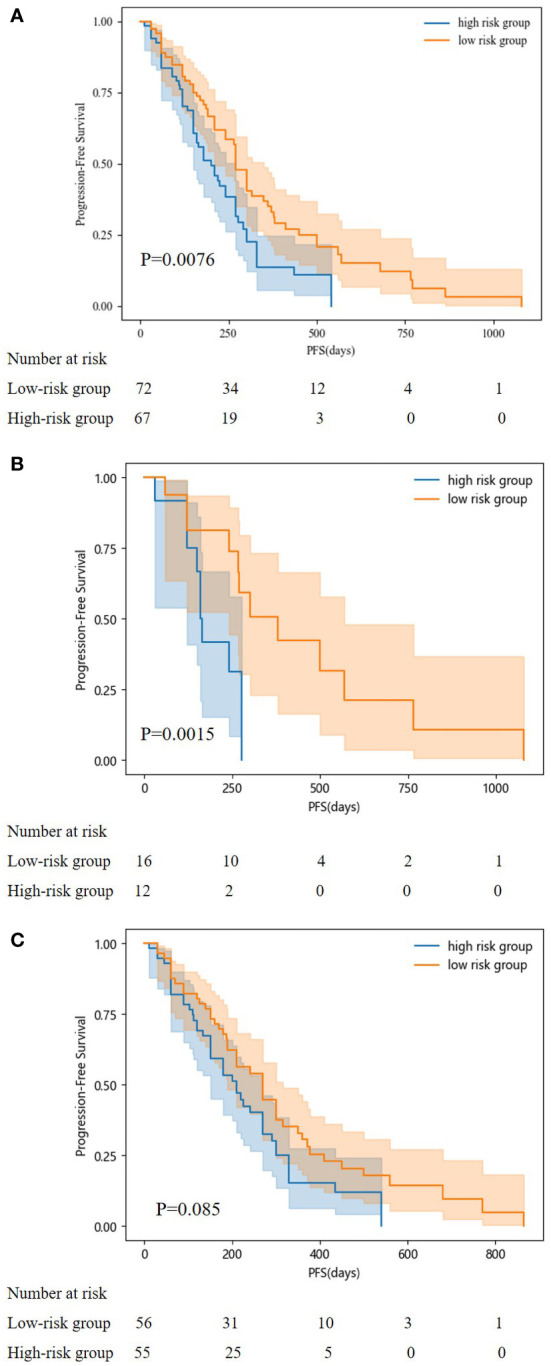
Kaplan-Meier curves for predicting PFS of patients in low and high-risk groups. (**A**: in the whole set; **B**: in testing set; **C**: in training set).

According to our PFS prediction model, FIGO stage, CCR, activated partial thromboplastin time (APTT), globulin (GLO), direct bilirubin (DBIL), FFA, blood urea nitrogen (BUN), HDL-C, and TG were independent predictors for EOC patients (R^2^ scores of different features are listed in **Table 3**), and they served as the foundation for our nomogram. The nomogram was created to predict the patients’ 1-year and 2-year PFS based on the independent prognostic variables (**Figure 4**). The patients in our study were divided into two groups based on their total scores on the nomogram as follows: the low-risk group (total score < 298) and the high-risk group (total score ≥ 298). Between the two distinct risk categories, there was a sizable variation in PFS in the whole set and the testing set (**Figures 5A, B**). In the training set, the median PFS in the low-risk group was longer, yet, the difference appeared to be negligible (**Figure 5C**). Compared with the high-risk group, the median PFS in the low-risk group was much longer (343 vs. 223 days) in the whole set. According to Kaplan–Meier curves of the whole set, the 1-year PFS rates for the low- and high-risk groups overall were 34.9% and 13.5%, respectively, and the 2-year PFS rates were 9.1% and 0%, respectively.

**Table 3:** Coefficients of different features.

Code name	R^2^ score
CCR	0.078452
APTT	-0.152512
FIGO	0.360724
GLO	0.566917
DBIL	0.648283
FFA	0.716480
BUN	0.750900
HDL-C	0.760655
TG	0.790584

## Discussion

EOC is a highly aggressive malignancy of the ovarian tissues. The prognosis of EOC patients is dependent on well-established prognostic criteria such as disease stage at diagnosis, disease recurrence, and treatment resistance. Despite the reported efficacy of bevacizumab in improving PFS, the optimal timing and duration of its use, cost-effectiveness, and identification of biologic markers that best predict its outcomes remain unclear (21). Therefore, there is a need for further research that focuses on identifying potential biomarkers to expand the indications for bevacizumab and improve patient selection for its use.

In this context, we conducted a retrospective study that investigated the changes in serum lipid levels in 139 EOC patients receiving bevacizumab treatment. Our findings indicate that bevacizumab treatment increases the levels of TC, TG, APOA1 and FFA while reducing APOB. These changes may be attributed to tumor hypoxia induced by bevacizumab, which triggers the metabolic reprogramming of fatty acid oxidation (22). Several studies have reported that advanced cancers exhibit elevated levels of lipogenic enzymes such as fatty acid synthase (FASN) (23). Increased FASN levels and fatty acid *de novo* synthesis have been observed in various fatty tissue-rich tumors, including breast (24), gastric (25), prostate (26), and ovarian (27) cancers. Upregulated FASN levels have been linked to increased fatty acid production and poor prognosis in several malignancies (28). Moreover, fatty acid β-oxidation, which is essential for signaling and energy transduction, allows EOC cells to retain high amounts of adenosine triphosphate (ATP), a critical component of cellular energy production. Excessive amounts of ATP have been linked to numerous physiological and pathological diseases over time, including infection (29), inflammation (30), and cancer (31). Therefore, anti-FASN and statin medications along with anti-VEGF medications may be combined to improve the prognosis for EOC patients receiving bevacizumab treatment.

The prognosis of EOC patients undergoing bevacizumab treatment has been challenging to predict with high precision. Clinical decision-making for EOC patients undergoing bevacizumab treatment can be improved with a good PFS prediction. To the best of our knowledge, based on our method employing machine learning techniques, this is the first model that is able to predict PFS in EOC patients receiving bevacizumab treatment. Our model might offer clinicians a simple-to-use prediction tool when treating patients with EOC. To predict these patients’ PFS, Light GBM classifier, XGB classifier, HGBoost Classifier, and Extra-Trees models were created. The outcomes demonstrated that the Extra-Trees model performed better than all other models in predicting cancer deaths, recurrences, and survival months. The Extra-Trees model was ultimately selected as our PFS prediction model. The AUC score of the model was 0.86, and the calibration curve was the closest to the diagonal. The difference between the data set’s samples and the model’s predictions, or R^2^ score, is one of the performance evaluation metrics for regression-based machine learning models. If the R^2^ score is 1, the model is perfect; if it is zero or even lower, the model performs poorly on a data set that has not yet been observed. This means that the model has been trained to perfection if the R^2^ score value is close to 1. The PFS of EOC patients receiving bevacizumab treatment can be predicted based on the R^2^ score of our model trained here, which is 0.790584.

In this study, we used our model to analyze the baseline characteristics of the patients and then created a prognostic nomogram to predict the 1-year and 2-year PFS of the patients. This nomogram may be useful for prognostic evaluation, the choice of treatment approach, and the follow-up management of these patients. According to our model, a number of variables, such as the FIGO stage, CCR, APTT, GLO, DBIL, FFA, BUN, HDL-C, and TG, had a substantial impact on PFS. Our findings on FIGO stage are consistent with analysis from MITO-16A/MaNGO-OV2A (32). In addition, our study confirms that CCR, APTT, GLO, DBIL, FFA, BUN, HDL-C, and TG are prognostic factors. These findings can be explained that patients have a worse prognosis when they are at a higher FIGO stage at diagnosis (33), and some earlier research has suggested that elevated levels of fatty acids are linked to an increased risk of cancer development because they control a number of biological processes, including maintaining the structure of cancer cell membranes and transmitting oncogenic signals (34, 35). Bevacizumab is known to induce intratumoral hypoxia, which in turn triggers the metabolic reprogramming of fatty acid oxidation. This process enables EOC cells to maintain high levels of ATP, which serves as a signaling messenger by providing the necessary carbon source for endothelial cells to synthesize DNA (36). Consequently, this may promote the growth of ovarian cancer and lead to the development of resistance against bevacizumab. Coagulation, liver, and renal functions—referred to APTT, GLO, and BUN—could affect a drug’s pharmacokinetics (37) and reduce PFS. Therapeutic judgments could be aided by these findings.

The Kaplan–Meier curves for progression-free survival (PFS) in patients of the two risk groups were plotted according to the cutoff value determined by the overall score on the nomogram. Patients in the low-risk category had higher PFS probabilities. The difference in the training set appeared to be negligible; yet, the median PFS in the low-risk group was still longer. The median PFS for the low-risk group in our study was 343 days, which was shorter than the median PFS for the earlier clinical studies. GOG-218 (8), MITO-16A/MaNGO-OV2A, and the high-risk group in the ICON-7 trial (7). These studies reported median PFS of 14.1 months, 20.7 months, and 15.9 months, respectively. The partially distinct populations included in these studies may explain the disparity in the median PFS.

Although our study has certain limitations, such as its retrospective nature and lack of external validation for the models created, the Extra Tree model appears to have the best internal validation. Additionally, the data used in this study may not be representative of populations in other regions due to its reliance on the authors’ institution, which could lead to discrepancies in results. Further research is necessary to confirm the results of this study through external validation and the inclusion of data from a wider variety of sources.

## Data availability statement

The original contributions presented in the study are included in the article/**Supplementary Material**. Further inquiries can be directed to the corresponding author.

## Ethics statement

The studies involving human participants were reviewed and approved by Medical Ethics Committee of the First Affiliated Hospital of Anhui Medical University. Written informed consent for participation was not required for this study in accordance with the national legislation and the institutional requirements.

## Author contributions

PL contributed to the idea and manuscript revision. XH collected and analyzed the data, drew the figures and tables and wrote the manuscript. YH contributed to manuscript revision. All authors contributed to the article and approved the submitted version.
